# Integration of gene co-expression analysis and multi-class SVM specifies the functional players involved in determining the fate of HTLV-1 infection toward the development of cancer (ATLL) or neurological disorder (HAM/TSP)

**DOI:** 10.1371/journal.pone.0262739

**Published:** 2022-01-18

**Authors:** Mohadeseh Zarei Ghobadi, Rahman Emamzadeh

**Affiliations:** Department of Cell and Molecular Biology and Microbiology, Faculty of Biological Science and Technology, University of Isfahan, Isfahan, Iran; Chinese Academy of Sciences, CHINA

## Abstract

Human T-cell Leukemia Virus type-1 (HTLV-1) is an oncovirus that may cause two main life-threatening diseases including a cancer type named Adult T-cell Leukemia/Lymphoma (ATLL) and a neurological and immune disturbance known as HTLV-1 Associated Myelopathy/Tropical Spastic Paraparesis (HAM/TSP). However, a large number of the infected subjects remain as asymptomatic carriers (ACs). There is no comprehensive study that determines which dysregulated genes differentiate the pathogenesis routes toward ATLL or HAM/TSP. Therefore, two main algorithms including weighted gene co-expression analysis (WGCNA) and multi-class support vector machines (SVM) were utilized to find major gene players in each condition. WGCNA was used to find the highly co-regulated genes and multi-class SVM was employed to identify the most important classifier genes. The identified modules from WGCNA were validated in the external datasets. Furthermore, to find specific modules for ATLL and HAM/TSP, the non-preserved modules in another condition were found. In the next step, a model was constructed by multi-class SVM. The results revealed 467, 3249, and 716 classifiers for ACs, ATLL, and HAM/TSP, respectively. Eventually, the common genes between the WGCNA results and classifier genes resulted from multi-class SVM that also determined as differentially expressed genes, were identified. Through these step-wise analyses, PAIP1, BCAS2, COPS2, CTNNB1, FASLG, GTPBP1, HNRNPA1, RBBP6, TOP1, SLC9A1, JMY, PABPC3, and PBX1 were found as the possible critical genes involved in the progression of ATLL. Moreover, FBXO9, ZNF526, ERCC8, WDR5, and XRCC3 were identified as the conceivable major involved genes in the development of HAM/TSP. These genes can be proposed as specific biomarker candidates and therapeutic targets for each disease.

## Introduction

Human T-cell Leukemia Virus type-1 (HTLV-1) is a human deltaretrovirus, which develops a lifelong infection [1]. The HTLV-1 infection leads to developing two main diseases including Adult T-cell Leukemia/Lymphoma (ATLL) and HTLV-1 Associated Myelopathy/Tropical Spastic Paraparesis (HAM/TSP) in about 5% of the infected subjects, however, the majority of the infected individuals remain in an asymptomatic carrier (AC) state [2, 3]. HTLV-1 is an endemic virus in the East North of Iran, sub-Saharan Africa, the Caribbean region, South America, and Japan [4]. ATLL is the malignancy of mature CD4+ of activated T lymphocytes. The genetic variation in various tumor repressor genes including p16INK4B, p15INK4A, p19INK4D, p18INK4C, p27KIP, p21WAF1, p57KIP2, Rb, and p53 occurred in ATLL [5]. HAM/TSP is another disease caused by HTLV-1 which is specified by perivascular inflammatory infiltrates in the spinal cord and brain. The infiltrating CD8+ and CD4+ lymphocytes are existing in the inflammatory lesions of the spinal cord [6]. The main challenge regarding this virus is which functional players cause the separation of pathogenesis routes to each of the mentioned diseases.

Weighted gene co-expression network analysis (WGCNA) is a powerful algorithm that clarifies the correlation patterns among genes. It also determines the highly correlated (co-expressed) gene groups which possibly regulate similar biological pathways. The co-expression network may also be employed to find regulatory genes with different phenotypes [7, 8].

Machine-learning (ML) denotes a set of computational models and algorithms to classify the biological data and then to predict external data. ML methods also utilize feature selection approaches to identify a collection of more relevant features [9]. Support vector machine method is a powerful classification technique. In its most simple type, SVM performs binary classification and naturally classifies the data samples into two classes. For multiclass classification, it breaks down the problem into multiple binary classification problems. It is performed through mapping data points to high dimensional space to obtain reciprocal linear segregation between every two classes, which is called the one-to-one approach [10]. With a larger number of samples, the performance is poor. SVMs have excellent efficiency in generalization. However, in the test stage, they can be highly slow. SVM has high algorithmic complexity and extensive memory requirement due to the use of quadratic programming.

As mentioned above, the differential classifier genes that ultimately determine the progression of the HTLV-1 infection to ATLL as a virus-caused cancer type and HAM/TSP as a virus-caused neurologic disease has not been yet completely determined. Therefore, we employed WGCNA and multi-SVM classification methods to find the main regulators of each disease.

## Materials and methods

### Datasets, merging, and preprocessing

The Gene Expression Omnibus (GEO) repository database was explored to find the datasets related to ACs, ATLL, and HAM/TSP. A total of six microarray datasets including GSE29312 [11], GSE29332 [11], GSE55851 [12], GSE38537 [13], GSE33615 [14], and GSE82160 [15] were found for analysis and validation. The characteristic of each dataset is described in Table 1.

**Table 1:** Details of datasets included in the analysis and validation.

	Platform		
	Dataset	ACs	Number of Samples
**Train**	GSE29312	Illumina HumanHT-12 V3.0 expression beadchip	Normal: 9 ACs: 20
	GSE29332	Illumina HumanWG-6 v3.0 expression beadchip	Normal: 8 ACs: 17
**Test**	GSE38537	Agilent-014850 Whole Human Genome Microarray 4x44K G4112F	ACs: 4
	GSE55851	Agilent-026652 Whole Human Genome Microarray 4x44K v2	ACs: 6
		**ATLL**	
**Train**	GSE33615	Agilent-014850 Whole Human Genome Microarray 4x44K G4112F	ATLL: 52
**Validation and Test**	GSE55851	Agilent-026652 Whole Human Genome Microarray 4x44K v2	ATLL: 12
		**HAM/TSP**	
**Train**	GSE29312	Illumina HumanHT-12 V3.0 expression beadchip	HAM/TSP: 10
	GSE29332	Illumina HumanWG-6 v3.0 expression beadchip	HAM/TSP: 10
**Validation and Test**	GSE82160	Affymetrix Human Gene 1.0 ST Array	HAM/TSP: 6

**Fig 1:**
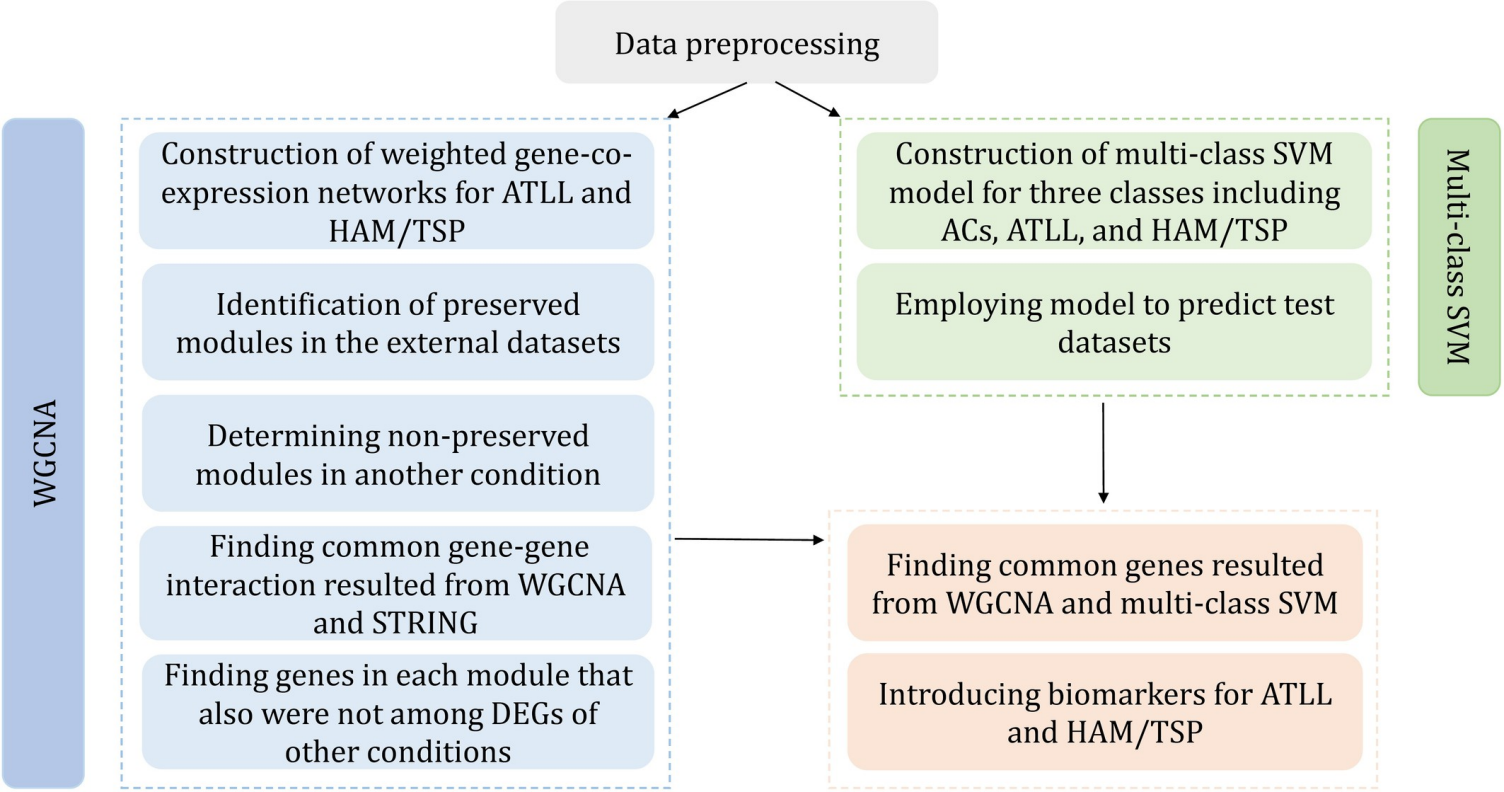
Workflow of the proposed method.

A total of 52 ATLL and 20 HAM/TSP samples were employed for the construction of weighted co-expression networks. The same samples of ATLL and HAM/TSP accompanied by 37 ACs were utilized to construct a classification model. Furthermore, a total of 10 ACs, 19 ATLL, and 6 HAM/TSP samples were utilized to validate WGCNA results and also as the test datasets for SVM analysis. In order to remove batch effect among datasets with different platforms, removeBatchEffect function in the limma package was applied [16]. The gene expression data belonging to each condition were merged, individually. A total of 7707 common genes were used for further analysis. The merged data were quantile-normalized and log2-transformed. Fig 1 represents the workflow of the used procedures to identify the major genes involved in the progression of ATLL and HAM/TSP.

### Weighted gene co-expression network analysis (WGCNA)

In order to find the co-expression genes and networks, WGCN analysis was performed by employing the WGCNA package in R [17]. Briefly, a similarity matrix containing Pearson correlation among all gene pairs was first calculated. Afterward, the scale-free topology fit index was calculated considering the soft-thresholding power β. The weighted adjacency matrix was then computed by raising the elements of the similarity matrix to the power β and adjusting the parameters as follows: type = "signed", corFnc = "bicor". Next, a topological overlap matrix (TOM) comprising the value of gene network connectivity was built. The dynamic hybrid tree cutting algorithm was applied to obtain the modules. The dynamic hybrid tree cutting algorithm was then utilized to determine the modules through cutting the obtained hierarchical clustering with the “hclust” function. Finally, the neighbor clusters were merged and final gene groups were determined.

### Module preservation

To assess the conservation of identified modules in the external datasets and other conditions, module preservation analysis was utilized. To this end, the function of “modulePreservation” in the WGCNA package was applied to compute medianRank and Z_summary_ by a permutation test (200 times). Modules with a medianRank≤9 and *Z*_summary_>2 were considered as moderate-high preserved modules in the external datasets and vice versa [18, 19].

### Protein-protein interaction networks and enrichment analysis

In order to determine the interaction of identified genes in the preserved modules at the protein level, protein-protein interaction networks (PPINs) were found using the STRNG database version 11.0. Furthermore, to find the enriched pathways by the identified genes, the KEGG database utilizing g:Profiler web tool (version: 1185_e69_eg16) was explored.

### Identification of differentially expressed genes

To identify the differentially expressed genes (DEGs) among normal vs. ACs (DEGs_NA), ACs vs. ATLL (DEGs_AA), and ACs vs HAM/TSP (DEGs_HA) the limma package in the R environment was applied. Benjamini-Hochberg FDR adjusted p-values < 0.05 and logFC = |0.8| were selected as a criterion for finding significant DEGs.

### Multi-class support vector machine

In order to determine the most important genes that classify ACs, ATLL, and HAM/TSP, a One-versus-One linear kernel multi-class SVM was performed using geNetClassifier package [20]. This method is based on the selection of genes through a wrapper forward feature selection and performing 8-fold cross-validation. For each cross-validation iteration, the training is primarily commenced by the prime rating genes of each class and assesses its performance. In each step, one gene is added if the accurate prediction is not obtained by the existing genes in that class. The number of genes applied to build the classifiers and the error of the classifiers are saved. The minimum number of classifier genes in each class that generated the classifier with a minimum error are chosen after running each cross-validation. The ultimate choice is carried out according to the selected genes in each cross-validation iterations. One of the most concerning issues in classification is the class imbalance because it leads to ignoring the class with the minimum members for the benefit of the class with the maximum members [21]. In this study, to overcome the imbalance between the number of samples of three classes, the SMOTE algorithm in Python was employed. SMOTE produces artificial samples for a class with a small number of objects. This algorithm is performed by considering the resemblance in the feature space among the existing objects utilizing the k-nearest neighbor algorithm (kNN algorithm) [22].

## Results

### WGCNA results

**Fig 2:**
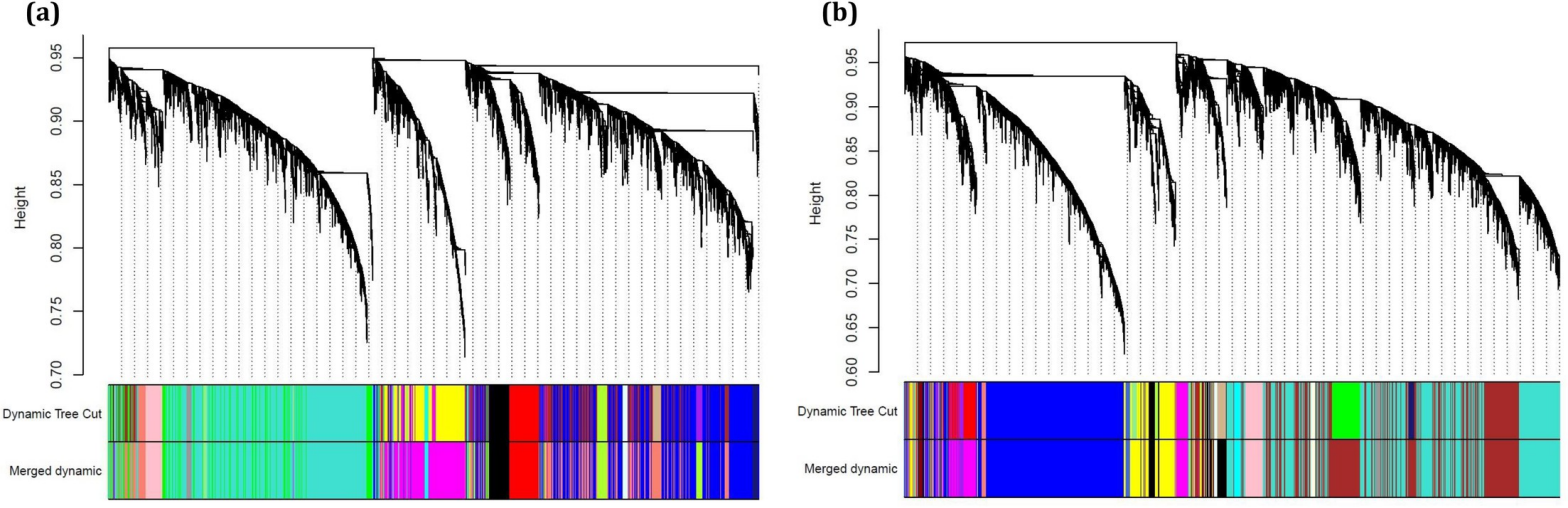
Dendrogram of clustered genes for (a) ATLL and (b) HAM/TSP based on a dissimilarity measure (1-TOM). The colors of rows represent the module membership resulted from the dynamic tree cut method and after merging modules.

In this study, two weighted gene co-expression networks were constructed for ATLL and HAM/TSP. For this purpose, a total of 52 ATLL and 20 HAM/TSP samples containing 7707 common unique genes were analyzed. The power β of 5 and 6 were obtained as the optimum soft-thresholding power for ATLL and HAM/TSP, respectively. After determining adjacency and TOM matrixes for each condition and then clustering the genes, the close clusters (modules) were then merged by adjusting the threshold value to 0.25. As a result, 14 and 15 modules were found for ATLL and HAM/TSP, respectively. Fig 2 represents the cluster dendrogram and identified modules before and after merging in which modules are specified by a unique color.

### Identification of specific modules for each condition

**Fig 3:**
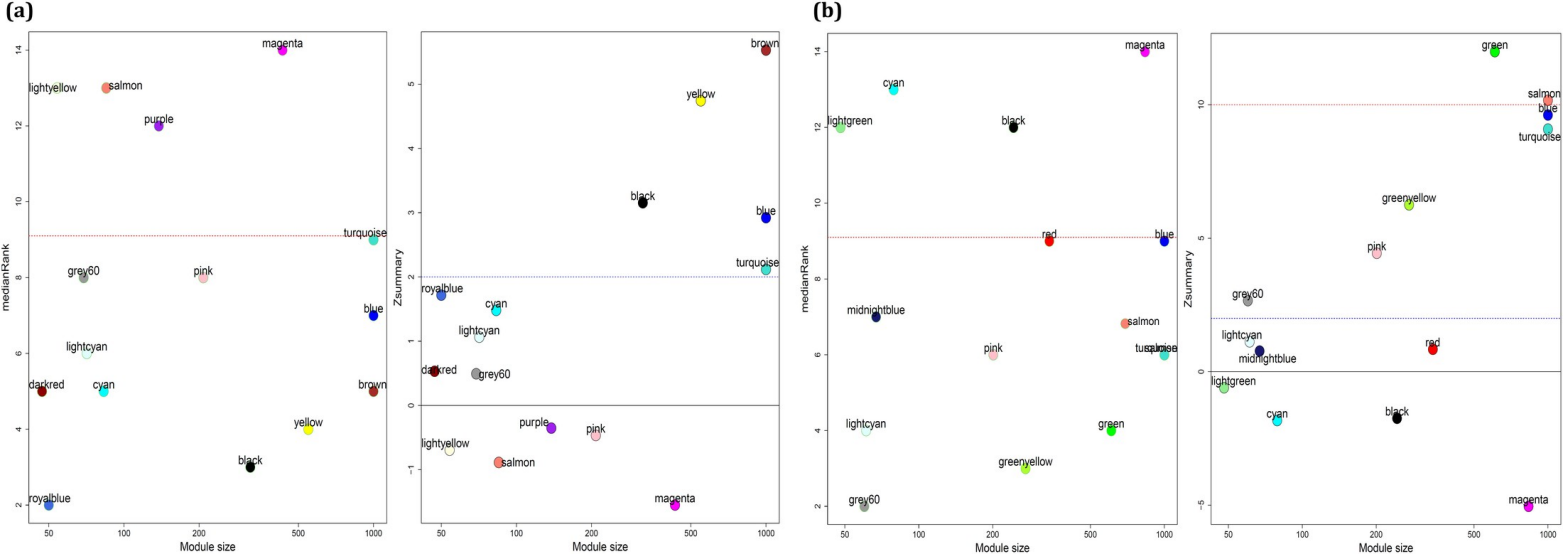
The medianRank and Z_summary_ preservations plots against module size for determining preserved modules of (a) HAM/TSP and (b) ATLL modules in the external dataset. Modules with a medianRank≤9 and *Z*_summary_>2 were considered as moderate-high preserved modules.

**Fig 4:**
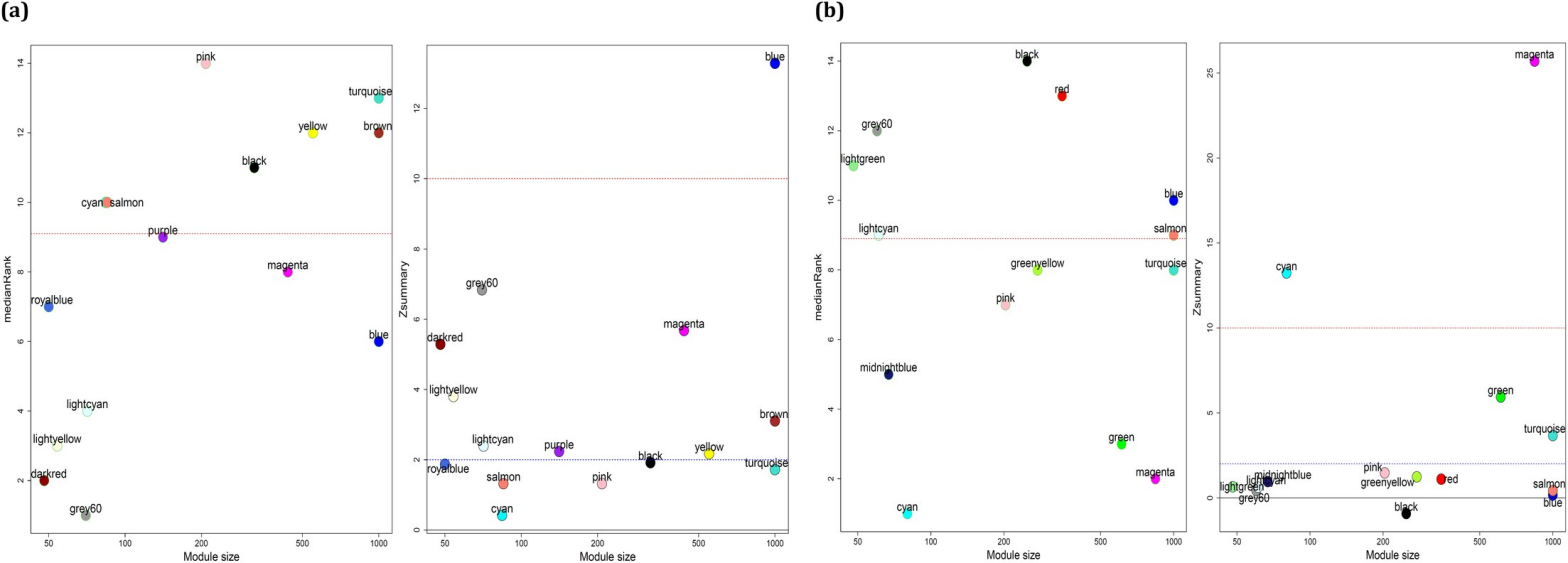
The medianRank and Z_summary_ preservations plots against module size for determining preserved modules of (a) HAM/TSP and (b) ATLL modules in ATLL and HAM/TSP datasets, respectively. Modules with a medianRank>9 and *Z*_summary_<2 were considered as non-preserved modules.

In this step, the identified modules for each condition were validated in the external datasets. Therefore, the preservation of ATLL modules in GSE55851, as well as preservation of HAM/TSP modules in GSE82160, were surveyed using “modulePreservation” function. The results showed the preservation of black, yellow, brown, blue, and turquoise modules in HAM/TSP, and grey60, greenyellow, green, pink, salmon, turquoise, blue modules in ATLL (Fig 3A and 3B). In this study, we aimed to find the specific gene players that help to progress the diseases in the HTLV-1 infected subjects from AC state to ATLL or HAM/TSP. Therefore, the specific modules for ATLL and HAM/TSP were determined through exploring the non-preserved modules of ATLL in HAM/TSP as well as the non-preserved modules of HAM/TSP in ATLL. The outcomes showed the non-preservation of grey60, salmon, and blue related to ATLL, and the non- preservation of black and turquoise modules related to HAM/TSP (Fig 4A and 4B). In order to investigate whether the co-expressed genes in each module were also had an interaction at the protein level, they were submitted to the STRING. The common interaction between correlated genes in modules and the interacted genes at the protein level was found. The results are mentioned in S1 Table. Moreover, the DEGs_NA, DEGs_AA, and DEGs_HA were identified (S2 Table). To ensure the selection of unique genes, the uncommon genes between the module members of ATLL with DEGs_NA and DEGs_HA and between the module genes of HAM/TSP with DEGs_NA and DEGs_AA were determined (S3 Table).

### Multi-class SVM

**Fig 5:**
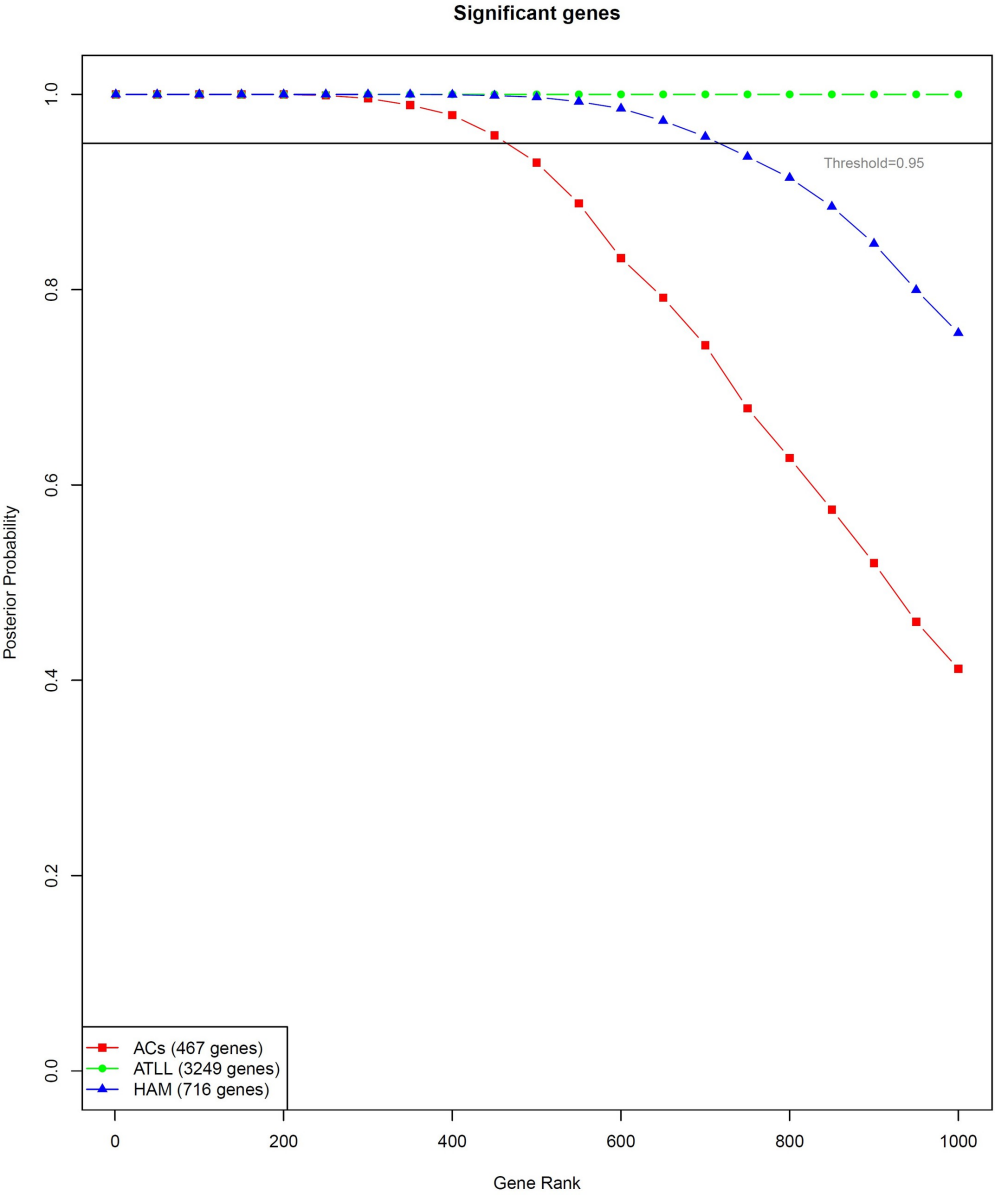
Graph representing the posterior probability of the top genes included in the gene ranking for ACs, ATLL, and HAM/TSP. Genes with posterior probability higher than the threshold (> 0.95) can be introduced as remarkable candidates to mark each condition.

The identification of genes associated with ATLL and HAM/TSP can shed light on the pathogenesis mechanisms of each disease. Therefore, multi-class SVM was executed to find classifier genes between ACs, ATLL, and HAM/TSP conditions. A number of 9 support vectors were constructed. The constructed models had an accuracy, sensitivity, and call rate of 100% for all classes except the call rate for ATLL which was 98.077. To do this, three conditions were compared versus each other using the empirical Bayes method performed using EBarrays package. This calculates a posterior probability for each gene to be differentially expressed in one of the conditions. Then, the genes were sorted by their probability and a gene ranking was performed by their statistical significance. As a result, 467, 3249, and 716 significant genes were found for ACs, ATLL, and HAM/TSP, respectively (Fig 5, S4 Table). The posterior probability matrix is mentioned in S5 Table. Moreover, the external validation was performed for the test datasets which are mentioned in Table 1. The posterior probability matrix of test classes is also mentioned in S5 Table.

### Determining specific genes in ATLL and HAM/TSP

The most important genes that classified the disease, were determined by exploring common genes between unique genes in each module and classifier genes identified by SVM. As a result, a total of 97 genes in salmon and 306 genes in blue modules of ATLL as well as 10 genes in black and 84 genes in turquoise modules of HAM/TSP were identified (S6 Table). The pathway enrichment analysis revealed the involvement of ATLL genes in immune and cancer pathways including NOD-like receptor signaling pathway, Pathways in cancer, Pathways of neurodegeneration—multiple diseases, Rap1 signaling pathway, Neurotrophin signaling pathway, GnRH signaling pathway, C-type lectin receptor signaling pathway, cAMP signaling pathway, Metabolic pathways, VEGF signaling pathway, PI3K-Akt signaling pathway, Apoptosis, Toxoplasmosis, p53 signaling pathway, Cell cycle, Proteoglycans in cancer, Chemokine signaling pathway, MAPK signaling pathway, Viral carcinogenesis, T cell receptor signaling pathway, Human T-cell leukemia virus 1 infection, Hippo signaling pathway, Cytokine-cytokine receptor interaction, NF-kappa B signaling pathway, JAK-STAT signaling pathway, FoxO signaling pathway, mTOR signaling pathway, Th17 cell differentiation, TNF signaling pathway, PD-L1 expression and PD-1 checkpoint pathway in cancer, Toll-like receptor signaling pathway, Pancreatic secretion, and TGF-beta signaling pathway. Moreover, the HMA/TSP genes were enriched in Pathways of neurodegeneration-multiple diseases, Huntington disease, Metabolic pathways, Prion disease, Alzheimer disease, Parkinson disease, Oxidative phosphorylation, Ubiquitin mediated proteolysis, TGF-beta signaling pathway, Cell cycle, N-Glycan biosynthesis, HIF-1 signaling pathway, MicroRNAs in cancer, Carbon metabolism, and AMPK signaling pathway which are mostly neurological related pathways. Among identified genes in this step, PAIP1, BCAS2, COPS2, CTNNB1, FASLG, GTPBP1, HNRNPA1, RBBP6, TOP1, SLC9A1, JMY, PABPC3, and PBX1 in ATLL have |logFC|≥0.8, and FBXO9, ZNF526, ERCC8, WDR5, and XRCC3 in HAM/TSP have |logFC|≥0.6. These genes can be introduced as possible specific biomarkers and therapeutic targets for each disease.

## Discussion

Despite various studies on the functional genes that have roles in developing ATLL or HAM/TSP in the asymptomatic carriers, the reports regarding the critical factors that differentiate the progression to each of these diseases are scarce. In this work, we tried to find the classifier genes that determine the fate of HTLV-1 infection.

Weighted gene co-expression network is a powerful approach to find the groups of genes with a high correlation that possibly regulate similar pathways in disease. WGCNA has been successfully being applied to identify co-regulated genes in HAM/TSP [18], influenza [23], hepatitis B-associated hepatocellular carcinoma [24], COVID-19 [25], and etc. Moreover, machine learning algorithms such as SVM have been widely applied to classify various virus-caused diseases based on classifier genes [26, 27]. SVM was also applied to predict the interaction between human proteins with human papillomaviruses and hepatitis C virus proteins [28] as well as viral subtyping classification [29]. The major disadvantages of SVM are its computational demands and its susceptibility to overfitting, appertaining to the adopted kernel [30]. Moreover, when the number of samples is much less than the number of features, SVM is more probably has poor performances [31]. Despite the excellent efficiency of SVMs, they may be highly slow in the test step. However, when there is a large number of features, the linear Kernel SVM results in better outcomes. Linear kernel SVM is an efficient and fast kernel function when the data is linearly separated [32]. Herein, we used a linear kernel SVM as the data was classified linearly and fast. Moreover, the performance parameters of the train and test sets were acceptable.

In this study, a careful analysis including weighted gene co-expression analysis and machine learning revealed 5 genes including FBXO9, ZNF526, ERCC8, WDR5, and XRCC3 for HAM/TSP, and 13 genes including PAIP1, BCAS2, COPS2, CTNNB1, FASLG, GTPBP1, HNRNPA1, RBBP6, TOP1, SLC9A1, JMY, PABPC3, and PBX1 in ATLL. In the following, we discuss the identified classifier genes for each condition.

According to previous studies, the mutation of several genes in ATLL cases has been determined. They have critical roles in the TCR/NF-κB signaling, including PRKCB, VAV1, and PLCG1 as well as IRF4 and CARD11 in NF-κB signaling [33, 34]. Moreover, mutations in CCR7 and CCR4 were detected in most ATLL cases which lead to truncation of the C-terminal cytoplasmic domain recognized to regulate several biological processes. Both receptors are extremely expressed in ATLL cells and likely implicated the infiltration of ATL into other organs [34].

In this research, the identified genes for ATLL are mainly involved in proliferation and tumor progression. PAIP1 encodes a protein that binds to PABP in order to modulate the initiation of translation and protein biosynthesis. This process is essential for protein synthesis during different diseases since any disturbance in this step may result in oncogenic transformation [35, 36]. The upregulation and functional role of PAIP1 in the progression of several cancers like pancreatic, gastric, and cervical cancers have been reported [37, 38]. The dysexpression of PAIP1 may lead to proliferation, metastasis, and development of cancer. Moreover, the overexpression of PAIP1 enhances VEGF expression and can promote tumor angiogenesis [38]. BCAS2 is a subunit of the prp19 complex, which has a critical function in mitotic initiation since its knockdown results in abnormal mitosis in addition to a reduction of invasion and migration of cancer cells and enhancing p53-induced apoptosis [39, 40]. Therefore, overexpression of BCAS2 can progress the proliferation and apoptosis in ATLL. RBBP6 is a retinoblastoma tumor suppressor protein that binds to many other proteins. It represses cellular proliferation. The upregulation of RBBP6 can result in cell cycle arrest, apoptosis, and tumorigenesis. RBBP6 promotes cell viability, proliferation, and migration through the JNK signaling pathway [41, 42]. PBX1 as a member of the TALE-class homeodomain family is an essential oncoprotein for various processes such as skeleton patterning, hematopoiesis, and organogenesis [43, 44]. The dysregulation of PBX1 has been reported for ovarian, prostate, and esophageal cancer [45]. *PBX1* is the direct downstream target gene of the NOTCH3 signaling pathway, which is necessary for ovarian cancer cell survival and proliferation. Therefore, it can be considered as one of the major factors in developing ATLL [46]. COPS2 is an important component of the COP9 signalosome complex, which contributes to various cellular processes, the regulation of the ubiquitin conjugation pathway, and also development of sepsis in patients with TNF-α rs1800629 A variant [47, 48]. COP9 has critical roles in the control of cell cycle, apoptosis, and signal transduction which results in carcinogenesis and cancer progression. The increase in the expression level of COPS2 has been reported that be connected to chromosome instability (CIN) [49]. CIN progresses cell-to-cell heterogeneity and affects the genome of cancer cells and tumor evolution [50]. TOP1 is a substantial nuclear enzyme that catalyzes the interchange of DNA double-helix between different topological states. TOP1 implicates DNA replication, RNA transcription, and also preserving genome stability by regulating the supercoiling state of DNA. The upregulation of TOP1 is associated with the proliferation of tumor cells [51]. *FASLG* belongs to the tumor necrosis factor superfamily, which contributes to apoptosis induction triggered by attaching to FAS. The FAS/FASLG signaling pathway is important for the regulation of the immune system. Alteration of FASLG pathway may lead to cancer development [52]. The overexpression of FASLG simplifies the progression of the tumor. *GTPBP1* encodes a protein belonging to the AGP11/GTPBP1 family of GTP-binding proteins. It is upregulated by interferon-gamma. GTPBP1 also regulates exosome-mediated mRNA degradation. Through interaction with DIS3 as a possible tumor suppressor, GTPBP1 can progress tumor progression in ATLL [53]. JMY is a cytoplasmic regulator of actin dynamics and nuclear p53/TP53-cofactor that increases p53/TP53 response by interaction with p300/EP300 and involves DNA damage. It also interacts with ubiquitous transcriptional co-activators of p300/CBP and various sequence-specific transcription factors, including hypoxia-inducible factor-1α (HIF-1α), and promotes cell invasion [54, 55]. SLC9A1 is an important protein in regulating signal transduction, cell migration, pH homeostasis, tumor growth, and cell volume [56]. The upregulation of *SLC9A1* is positively associated with the level of immune infiltration and prognosis of cancer [57].

Among identified genes for ATLL, *CTNNB1* and *HNRNPA1* are related to EMT transition. CTNNB1 is a part of the constituted proteins of adherens junctions (AJs). AJs are essential for the growth and preservation of epithelial cell layers through adjusting adhesion between cells. The upregulation of *CTNNB1* as an epithelial-mesenchymal transition (EMT)-related gene has a significant role in the regulation of cancer signaling [58]. *HNRNPA1* is a member of the hnRNP family, which suppresses splicing by blocking the assembly of the splicing complex and developing distal splice site selection [59]. it progresses tumor invasion by regulation of CD44v6. The knockdown of *HNRNPA1* induces a remarkable decline in cell viability [60]. The overexpression of HNRNPA1 can promote cell invasion by inducing EMT transition [61].

PABPC3 belongs to poly(A)-binding proteins (PABP) that control the stability of messenger RNA and the initiation of translation. The information about the function of this protein in ATLL progression and other cancers is scarce, however, it was reported as a driver gene in follicular thyroid cancer and Glioblastoma [62, 63].

The identified genes for HAM/TSP are mainly involved in neurological disturbances and other related diseases. FBXO9 belongs to the F-box protein family, which constitutes one of the subunits of the ubiquitin-protein ligase complex. The possible association of FBXO9 and degenerative disease phenotypes and also the involvement in the development of neuronal disorders have been reported [64]. However, its functional role in HAM/TSP should be further investigated. *ZNF526* gene in developing brains suggests a possible role of this protein during development. Moreover, the ZNF526 biallelic variants affect eyes and brains in a neurodevelopmental disorder that leads to severe microcephaly [65]. *ERCC8* is a gene located on chromosome 5q12.1 that encodes a Cockayne syndrome A (CSA) protein. It implicates repairing damaged DNA as well as XRCC3 as a member of the RecA/Rad51-related protein family [66]. The downregulation of *ERCC8* and *XRCC3* in HAM/TSP samples may help the progression of the disease, however, more precise studies are required. WDR5 participates in gene regulation, apoptosis, signal transduction, and cell cycle progression. It is known that viral infection leads to a decrease in the expression level of WDR5 in the nucleus as observed in HAM/TSP [67, 68].

Our study has some limitations. The co-expressed and classifier groups were identified through the analysis of a high-throughput microarray dataset by the computational methods. Therefore, further experimental validation can better assess the introduced genes. In this study, we integrated several datasets for each condition, however, the analysis of large sample groups improves the validity of the analysis. On the other hand, the SVM algorithm is not proper for large datasets. However, using a suitable feature selection method eliminates this limitation as we used in this study. Moreover, the incorrect selection of kernel type may result in an increase in error percentage.

## Conclusion

In conclusion, we employed a step-wise procedure containing weighted gene co-expression method and multi-class SVM to identify the major genes involved in the developing disease in the HTLV-1 infected subjects. The final results revealed the involvement of ATLL genes in tumor progression and cancer development, and the implication of HAM/TSP genes in creating neurological disturbances. These genes can be introduced as potential biomarkers and also therapeutic targets. Certainly, further studies must be performed to assess the identified genes in large datasets with different patient populations and ethnicities.

## Supporting information

S1 Table The common interaction between correlated genes in modules and the interacted genes at the protein level. (XLSX)

S2 Table List of DEGs. (XLSX)

S3 Table The specific gene members of ATLL and HAM/TSP. (XLSX)

S4 Table The classifier genes for ACs, ATLL, and HAM/TSP resulted from multi-class SVM analysis. (XLSX)

S5 Table The posterior probability matrix of train and test classes. (XLSX)

S6 Table The common genes between unique genes in each module and classifier genes identified by SVM. (XLSX)
